# H-Ferritin Produced by Myeloid Cells Is Released to the Circulation and Plays a Major Role in Liver Iron Distribution during Infection

**DOI:** 10.3390/ijms23010269

**Published:** 2021-12-27

**Authors:** Ana C. Moreira, Tânia Silva, Gonçalo Mesquita, Ana Cordeiro Gomes, Clara M. Bento, João V. Neves, Daniela F. Rodrigues, Pedro N. Rodrigues, Agostinho A. Almeida, Paolo Santambrogio, Maria Salomé Gomes

**Affiliations:** 1 i3S—Instituto de Investigação e Inovação em Saúde, Universidade do Porto, 4200-135 Porto, Portugal; Ana.S.Moreira@ibmc.up.pt (A.C.M.); tania.silva@ibmc.up.pt (T.S.); ana.c.gomes@i3s.up.pt (A.C.G.); clara.bento@i3s.up.pt (C.M.B.); jneves@ibmc.up.pt (J.V.N.); drodrigues@ibmc.up.pt (D.F.R.); prodrigu@ibmc.up.pt (P.N.R.); 2 IBMC—Instituto de Biologia Molecular e Celular, Universidade do Porto, 4200-135 Porto, Portugal; goncalo.mesquita@univ-lille.fr; 3 ICBAS—Instituto de Ciências Biomédicas Abel Salazar, Universidade do Porto, 4050-313 Porto, Portugal; 4 Programa Doutoral em Biologia Molecular e Celular (MCbiology), Instituto de Ciências Biomédicas Abel Salazar da Universidade do Porto, 4200-135 Porto, Portugal; 5 LAQV/REQUIMTE, Departamento de Ciências Químicas, Faculdade de Farmácia, Universidade do Porto, 4050-313 Porto, Portugal; aalmeida@ff.up.pt; 6 Division of Neuroscience, San Raffaele Scientific Institute, 20132 Milan, Italy; santambrogio.paolo@hsr.it

**Keywords:** ferritin, H-ferritin, ferroportin, infection, mycobacterium, mouse, liver, histopathology, iron tissue distribution

## Abstract

During infections, the host redistributes iron in order to starve pathogens from this nutrient. Several proteins are involved in iron absorption, transport, and storage. Ferritin is the most important iron storage protein. It is composed of variable proportions of two peptides, the L- and H-ferritins (FTL and FTH). We previously showed that macrophages increase their expression of FTH1 when they are infected in vitro with *Mycobacterium avium*, without a significant increase in FTL. In this work, we investigated the role of macrophage FTH1 in *M. avium* infection in vivo. We found that mice deficient in FTH1 in myeloid cells are more resistant to *M. avium* infection, presenting lower bacterial loads and lower levels of proinflammatory cytokines than wild-type littermates, due to the lower levels of available iron in the tissues. Importantly, we also found that FTH1 produced by myeloid cells in response to infection may be found in circulation and that it plays a key role in iron redistribution. Specifically, in the absence of FTH1 in myeloid cells, increased expression of ferroportin is observed in liver granulomas and increased iron accumulation occurs in hepatocytes. These results highlight the importance of FTH1 expression in myeloid cells for iron redistribution during infection.

## 1. Introduction

Iron is essential for almost every living organism, as it plays key roles in DNA and RNA synthesis, mitochondrial respiration, and cell proliferation and differentiation, among other processes [1]. Nonetheless, iron can be dangerous due to its capacity to promote the generation of reactive oxygen species [2]. Iron homeostasis, including absorption, storage, and distribution, are thus essential and need to be tightly controlled.

Intracellular iron storage is mainly guaranteed by the highly conserved protein ferritin. Ferritin has a dual function: iron storage and cell protection against iron toxicity. Additionally, it has been increasingly recognized to have a role in immune regulation [3,4,5]. Mammalian cytosolic ferritin is a large multimeric spherical molecule, with 24 subunits, of two types of peptide chains: H-ferritin (FTH) and L-ferritin (FTL). The two types of ferritin subunits have nearly 20 kDa each but are coded at different gene loci. The 24-mer polypeptide forms an apoferritin shell, inside which more than 4000 Fe^3+^ atoms can be stored. FTH and FTL subunits are found at different ratios in the apoferritin shell, depending on the tissue. In 1991, the structure of a ferroxidase moiety on the FTH protein was found, disclosing the function of this subunit of ferritin [6]. The ferroxidase activity of FTH, not present in FTL subunits, is necessary for the conversion of ferrous to ferric iron, prior to enclosure and retention of the atoms inside the ferritin core. The essentiality of FTH is shown by the fact that mice deficient in the *Fth1* gene cannot survive embryonic development [7]. FTL, although not essential, is important for the incorporation of iron and for the stability of ferritin [8], contributing to long-term iron storage [9,10]. 

Infection and inflammation have a clear impact on iron homeostasis. Iron dysregulation may contribute to pathology, especially in the case of chronic or persistent infections [2]. In general, circulating iron levels decrease in response to the presence of microbial products. It is well established that LPS injection, for example, leads to hepatic production of hepcidin, which in turn will cause a decrease in the expression of ferroportin, decreased cellular iron export, and increased iron storage in myeloid cells [11,12]. Previous work from our group showed that different pathogens impact iron homeostasis through different mechanisms. Gram-negative bacteria such as *Salmonella* clearly induce hepcidin production, while Gram-positive pathogens such as *Listeria* and *Mycobacterium* may affect iron distribution by hepcidin-independent mechanisms, namely by directly affecting ferroportin expression [13].

Hyperferritinemia (increased level of serum ferritin) is one important inflammatory marker, although its contribution to pathology or infection resolution is not clear [10,14,15]. We previously showed that macrophages increase FTH1 expression when infected with *M. avium*, while FTL levels remain unaltered [16]. On the other hand, FTH1 expression modulates macrophage activation by immune and microbial stimuli and is essential for cell protection against oxidative stress [3,17], suggesting that FTH1 contributes to host defense against infection. Furthermore, Reddy et al. showed that mice deficient in FTH1 in cells of myeloid origin are more susceptible to infection by *M. tuberculosis* than wild-type mice [18].

In this work, we investigated the role of FTH1 in host protection against *M. avium* infection. We found that FTH1 of myeloid origin is released into the circulation and plays a crucial role in tissue iron redistribution during systemic *M. avium* infection. Overall, in our mouse model of infection, the deficiency in FTH1 in myeloid cells caused an increased resistance against *M. avium*, related to the restriction of pathogen access to iron.

## 2. Results

### 2.1. Mice Lacking FTH1 in Myeloid Cells Are More Resistant to M. avium Infection

**Figure 1:**
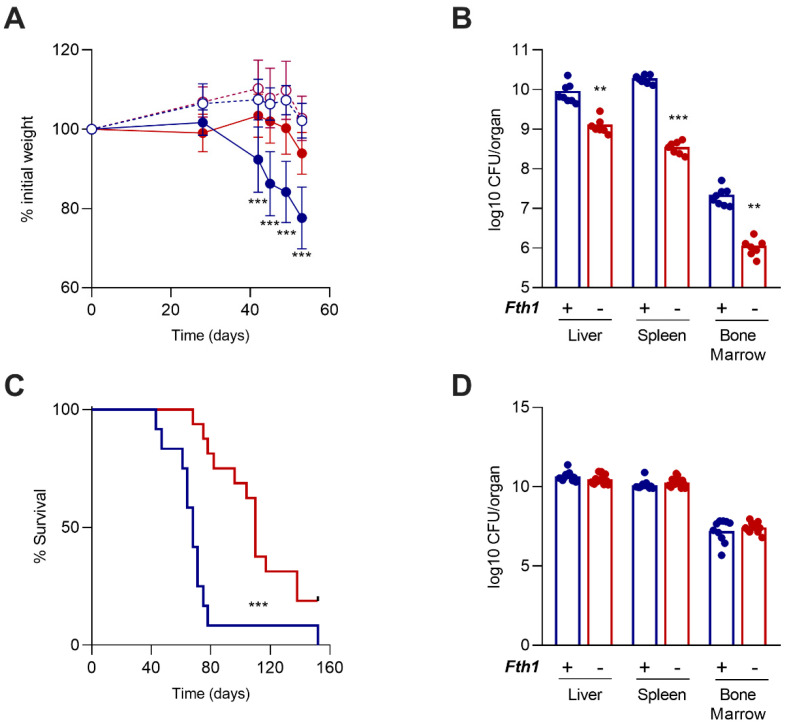
Evolution of infection by *Mycobacterium avium* in mice deficient in H-ferritin in myeloid cells. *Fth1^+/+^* (blue) and *Fth1^−/−^* (red) mice were intravenously infected with 10^6^ CFU of *M. avium* 25291. (**A**) Animals’ body weight throughout the experiment. Data are expressed as percentage of the initial weight, presented as the mean ± SD of 5 to 8 animals per group. Empty circles: noninfected; filled circles: infected. (**B**) Bacterial burden 60 days post-infection in the liver, spleen, and bone marrow of 8 *Fth1^+/+^* and 7 *Fth1*^−/−^ mice. Bars represent the mean, and circles represent each animal. (**C**) Kaplan–Meier survival curve corresponding to 12 *Fth1^+/+^* and 16 *Fth1*^−/−^ animals. Mice were euthanized when they lost 20% of the initial weight (between day 43 and day 152 post infection). (**D**) Bacterial burden in the liver, spleen, and bone marrow, at the time of euthanasia in the survival curve, of 12 *Fth1^+/+^* and 16 *Fth1*^−/−^ mice. Bars represent the mean and circles represent each animal. Statistics: multiple *t*-test in (**A**,**B**,**D**); log-rank (Mantel–Cox) test in (**C**). ** *p* < 0.01 and *** *p* < 0.001 when comparing *Fth1^−/−^* with *Fth1*^+/+^ mice.

Mice deficient in FTH1 production by myeloid cells (*Fth1^−/−^* mice) were intravenously infected with *M. avium* 25291, in parallel with *Fth1*^+/+^ littermates. *Fth1^−/−^* mice lost weight more slowly than *Fth1^+/+^* animals (Figure 1A). Furthermore, bacterial loads in the liver, spleen, and bone marrow, 60 days after infection, were significantly lower in *Fth1^−/−^* as compared to *Fth1^+/+^* mice (Figure 1B). Moreover, when we performed a survival curve, for which animals were euthanized whenever they had lost 20% of their initial weight, *Fth1^−/−^* mice survived longer than *Fth1^+/+^* (Figure 1C). Of note, three *Fth1^−/−^* animals did not reach the 20% loss in body weight 152 days post infection, when the experiment was terminated. Interestingly, in this experiment, the bacterial loads at the time of euthanasia were similar in mice with both genotypes (Figure 1D). These results suggest that the resistance of *Fth1^−/−^* mice is due to a slower growth of the mycobacteria. Similar results were obtained when *Fth1^−/−^* and *Fth1^+/+^* mice were intravenously infected with a less-virulent strain of *M. avium,* 2447 SmT (Figure S1A), and when mice were infected with *M. avium* 25291 via aerosol route (Figure S1B). In all these models, *Fth1^−/−^* mice were more resistant to mycobacterial infection than *Fth1*^+/+^ littermates.

### 2.2. Mice Deficient in FTH1 in Myeloid Cells Have a Less-Pronounced Inflammatory Response after Infection

**Figure 2:**
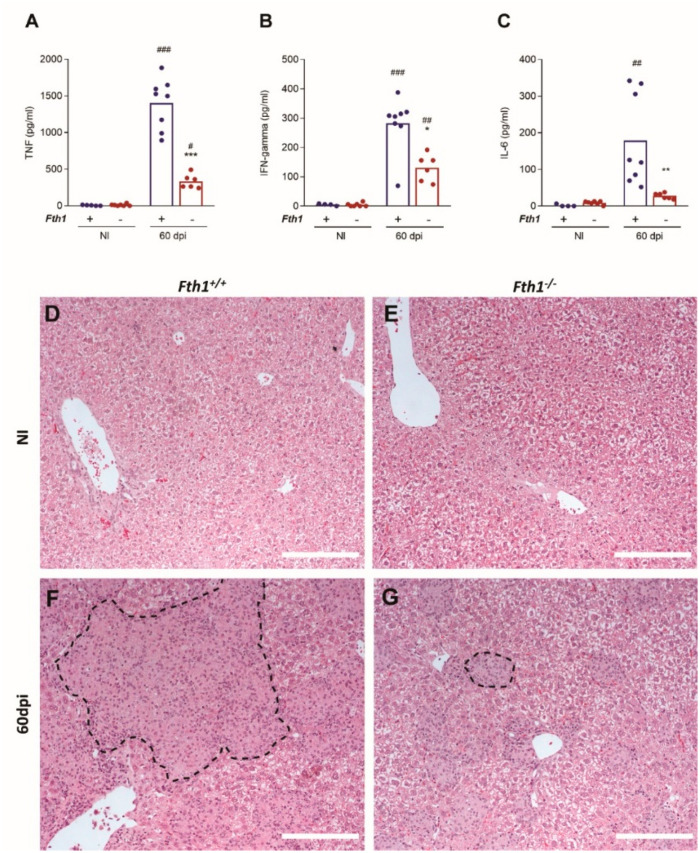
Inflammatory response in mice infected with *M. avium*. *Fth1^+/+^* (blue) and *Fth1^−/−^* (red) mice were intravenously infected with 10^6^ CFU of *M. avium* 25291 and euthanized 60 days later. (**A**) TNF-alpha, (**B**) IFN-gamma, and (**C**) IL-6 were measured in the serum, using a cytometric bead array kit. Bars represent the mean, and the circles represent individual values of 4 to 8 animals per group. Statistics: two-way ANOVA followed by Sidak multiple-comparison post hoc test. * *p* < 0.05, ** *p* < 0.01, *** *p* < 0.001 when comparing *Fth1^+/+^* to *Fth1^−/−^* mice. ^##^ *p* < 0.01, ^###^ *p* < 0.001 when comparing infected to noninfected mice of the same genotype. (**D**–**G**) Representative images of H&E-stained liver sections from noninfected (NI) and infected (60 dpi) *Fth1^+/+^* and *Fth1^−/−^* mice. Representative granulomas are outlined. Scale bar: 200 µm.

To investigate the causes of the increased resistance of *Fth1*^−/−^ mice, we analyzed several parameters of the immune response to infection. First, we measured the serum levels of proinflammatory cytokines. *Fth1^−/−^* mice had lower levels of circulating tumor necrosis factor (TNF)-alpha, interferon-gamma (IFN-gamma) and interleukin (IL)-6 than *Fth1*^+/+^ mice, 60 days after infection (Figure 2A–C). Of note, these decreased levels of proinflammatory cytokines were not due to a lower bacterial load, as they were also observed in serum samples collected in the survival curve experiment (Figure S2A–C), even though in that condition bacterial loads were similar between the two groups of mice (Figure 1D).

We also studied immune cell populations in the spleen, 60 days after intravenous infection. We did not find major differences in the number of macrophages, neutrophils, NK, and T cells between *Fth1*^−/−^ and *Fth1^+/+^* mice. The only significant difference we found was a higher number of CD19^+^ cells in *Fth1^−/−^* than in *Fth1^+/+^* mice (Figure S2D–I).

Mycobacterial infections are characterized by the formation of tissue granulomas. In our model of intravenous infection, liver granulomatous lesions are particularly evident at 60 days post-infection. The liver is also the organ where most of the bacteria are found throughout the infection and where the differences in bacterial load between *Fth1^−/−^* and *Fth1*^+/+^ mice were more pronounced. For that reason, we focused on the study of this organ. *Fth1*^−/−^ mice tended to have a lower percentage of tissue area occupied by granulomas, as compared to *Fth1*^+/+^ mice (39.8 ± 10.4% vs. 50.3 ± 13.3%) (Figure 2D–G), consistent with a decreased inflammatory response.

### 2.3. FTH1 Produced by Myeloid Cells Is Necessary for Liver Iron Storage and Infection-Induced Hypoferremia

**Figure 3:**
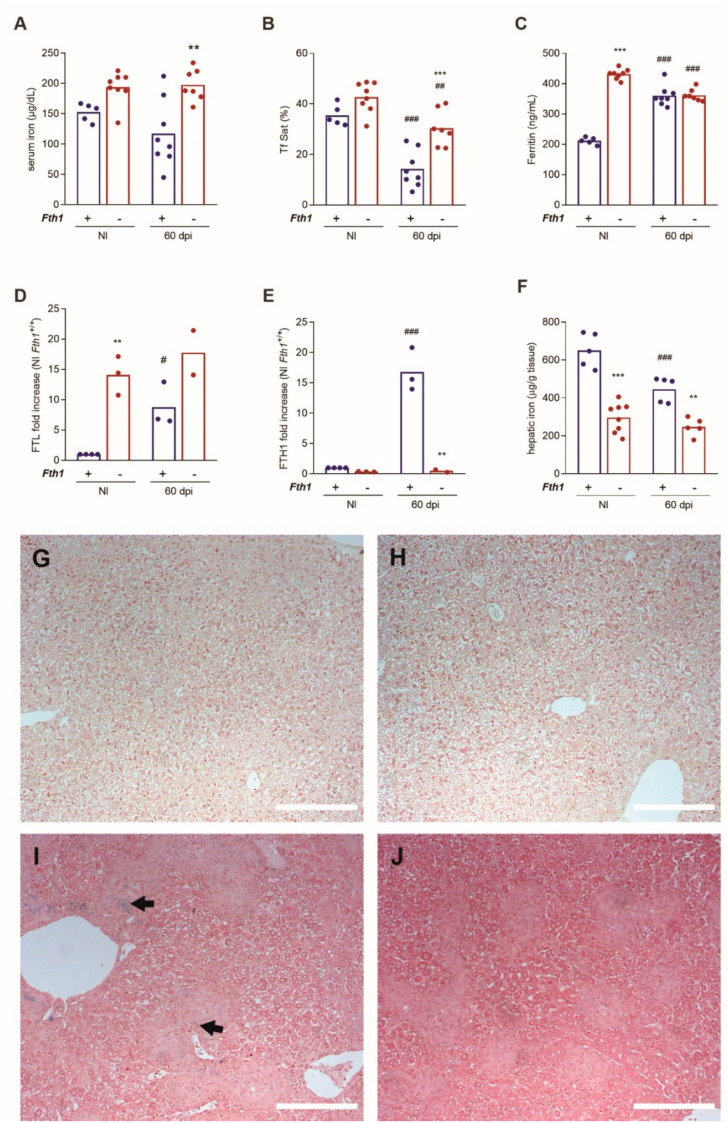
Impact of infection on iron distribution. Sera from *Fth1*^+/+^ (blue) and *Fth1*^−/−^ (red) mice infected for 60 days with *M. avium* 25291 (60 dpi) or from noninfected mice (NI), were used to measure (**A**) iron, (**B**) transferrin saturation, and (**C**) total ferritin (*n* = 5 to 8). (**D**,**E**) Serum FTL and FTH1 subunits were measured by ELISA (*n* = 2 to 4). (**F**) Liver sections were collected, and the iron concentration was determined by atomic absorption spectrometry (*n* = 5 to 8). Bars represent the mean, and circles represent each animal. Statistics: two-way ANOVA followed by Sidak multiple-comparison post hoc test. ** *p* < 0.01, *** *p* < 0.001 when comparing *Fth1^−/−^* with *Fth1*^+/+^ mice in the same experimental condition. ^#^ *p* < 0.05, ^##^ *p* < 0.01, ^###^ *p* < 0.001 when comparing infected to noninfected mice of the same genotype. (**G**–**J**) Perls’ staining of liver sections was used to evaluate iron distribution. Arrows indicate regions of iron accumulation (blue staining). Scale bar = 200 µm.

Evaluation of immune response parameters did not reveal any differences that could explain the increased resistance of *Fth1*^−/−^ mice. We next asked whether this resistance was associated with alterations in iron distribution. Serum iron levels in noninfected mice were not significantly different between the two genotypes (Figure 3A). Sixty days after *M. avium* infection, *Fth1^+/+^* mice had a significant decrease of transferrin saturation, in parallel with a tendency towards decreased serum iron, when compared to noninfected mice of the same genotype (Figure 3A,B). In *Fth1^−/−^* mice, the decrease in transferrin saturation and serum iron upon infection were less pronounced. Thus, when comparing infected mice, both serum iron and transferrin saturation were significantly higher in *Fth1*^−/−^ than in *Fth1*^+/+^ animals (Figure 3A,B). Regarding ferritin, *Fth1^−/−^* mice had higher serum levels in basal conditions, compared to *Fth1^+/+^* mice (Figure 3C), as previously reported [19]. With infection, serum ferritin was significantly increased in *Fth1^+/+^* mice while a small, but significant, decrease of serum ferritin was observed in *Fth1^−/−^* mice (Figure 3C). Although it is usually assumed that serum ferritin is formed solely of FTL chains, we decided to investigate whether this was the case in *M. avium*-infected mice. For that, we quantified each of the ferritin peptides in serum samples, using immune rabbit sera specific for each of the ferritin chains [16]. Surprisingly, we found that the increase in serum ferritin observed in *Fth1*^+/+^ mice upon infection was due to increases in both FTL and FTH1 chains (Figure 3D,E and Figure S3). Interestingly, no alterations of FTH1 serum levels were observed in *Fth1*^−/−^ mice, indicating that serum FTH1 originated from myeloid cells (Figure 3D,E and Figure S3). 

Given that serum iron indicators revealed important differences between *Fth1*^−/−^ and *Fth1*^+/+^ mice, we decided to investigate whether tissue iron levels were also different between the two groups of mice. We focused on the liver, which is the main target of this infection model and where the differences in bacterial load between the two genotypes were most evident. Before infection, *Fth1*^−/−^ mice had roughly half the concentration of liver iron found in *Fth1*^+/+^ mice (Figure 3F). Upon infection, iron concentration in the liver slightly but significantly decreased in both groups, remaining lower in *Fth1*^−/−^ than in *Fth1*^+/+^ mice (Figure 3F). The total amount of iron per organ followed the same pattern: before infection, *Fth1^+/+^* mice had 195.2 ± 93.95 µg of iron per liver, while *Fth1*^−/−^ had 80.6 ± 22.27 µg per liver. After infection, total liver iron in *Fth1^+/+^* mice was 108.8 ± 87.21 and in *Fth1*^−/−^ mice was 80.46 ± 38.49 µg. In accordance with these determinations, when liver sections were stained using the Perls’ technique, iron deposits were rarely seen, but were even rarer in *Fth1*^−/−^ mice, as compared to their *Fth1*^+/+^ littermates (Figure 3G–J).

Overall, these data indicate that iron redistribution in response to infection is altered in mice deficient in myeloid FTH1, with a tendency for increased iron in circulation and lower iron accumulation in the liver.

### 2.4. Iron Overload Reverts the Resistance of Fth1^−/−^ Mice, Indicating That Resistance Is Related to Iron Withholding

**Figure 4:**
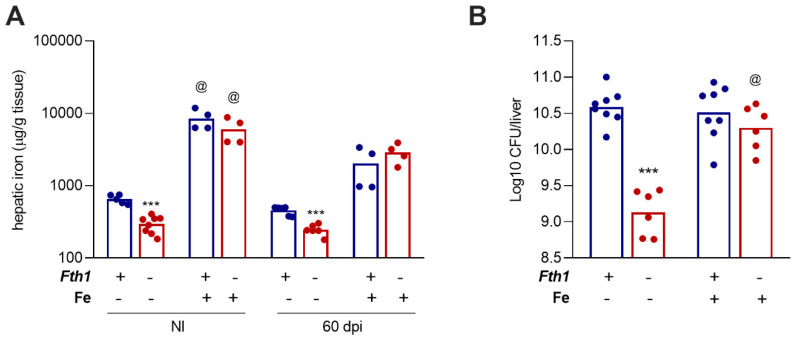
Effect of iron overload on the growth of *M. avium* in the liver. Groups of *Fth1^+/+^* (blue) and *Fth1^−/−^* (red) mice were intraperitoneally injected with 10 mg of iron, in the form of iron–dextran, 10 days before intravenous infection with 10^6^ CFU of *M. avium* 25291. Sixty days after infection, noninfected (NI) and infected (60 dpi) mice were euthanized. (**A**) Liver iron was quantified by atomic absorption spectrometry (*n* = 4 to 8 per group). (**B**) Bacterial burden in the livers, 60 days after infection (*n* = 6 to 8 per group). The bars represent the mean of the group, and each circle corresponds to one animal. Statistics: two-way ANOVA followed by Sidak multiple-comparison post hoc test. *** *p* < 0.001 when comparing *Fth1^−/−^* to *Fth1^+/+^* mice in the same experimental condition. ^@^ *p* < 0.001 when comparing iron-overloaded with non-iron-overloaded mice of the same genotype and infection status.

Based on the previous results, we hypothesized that the increased resistance of *Fth1*^−/−^ mice to infection might be due to the lower iron availability in the tissues, which could be limiting the *M. avium* growth. To test this hypothesis, we designed an experiment in which animals were iron-overloaded by intraperitoneal injection of iron–dextran (10 mg of iron/animal) ten days before infection. With this treatment, liver iron levels became similar between *Fth1*^+/+^ and *Fth1*^−/−^ mice (Figure 4A). While in non-iron-treated mice, the bacterial load in *Fth1*^+/+^ was about 10 times higher than in *Fth1*^−/−^ mice, after iron overload, the bacterial load in the liver was similar between the two genotypes (Figure 4B). These data suggest that the intrinsic resistance of *Fth1^−/−^* mice to *M. avium* infection was due to the lower levels of iron available in tissue for bacterial growth, highlighting the importance of FTH1 of myeloid origin for the regulation of iron storage and availability during infection. 

### 2.5. FTH1 of Myeloid Origin Affects Iron Redistribution upon Infection, through the Modulation of Ferroportin Expression

The results described above suggested that FTH1 produced by myeloid cells plays an important role in iron redistribution upon infection. In order to further identify the main players involved in iron redistribution, we measured the expression of iron-related genes in the liver. The expression of both *Fth1* and *Ftl* tended to increase in the liver in infected animals. The induction was more pronounced in *Fth1*^+/+^ as compared to *Fth1*^−/−^ mice (Table 1). However, none of these alterations was statistically significant. The infection led to a significant increase in the expression of heme-oxygenase-1 (*Hmox*), as observed in previous studies [20], independently of the genotype (Table 1). Interestingly, the expression of hepcidin (*Hamp1*) was also significantly increased in infected mice, which is in contrast with our previous work [21]. The induction of the expression of these two genes was higher in *Fth1*^+/+^ mice than in *Fth1*^−/−^ mice (Table 1). mRNA levels for ferroportin (*Slc40a1*) also tended to increase in infected, compared to noninfected, mice, irrespective of the genotype (Table 1).

**Figure 5:**
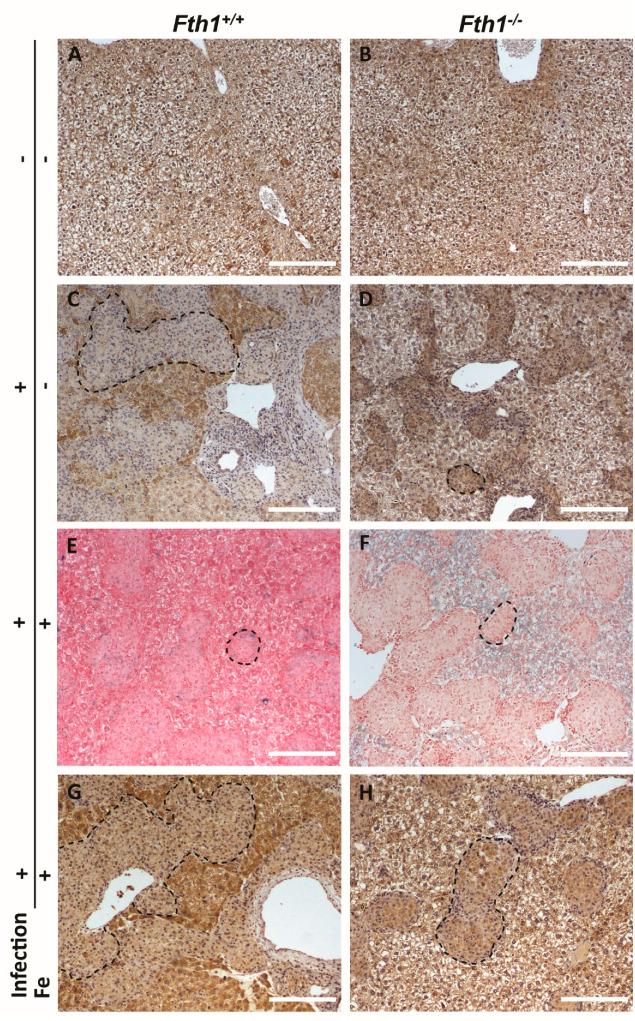
Effects of infection and myeloid *Fth1* deficiency in iron and ferroportin distribution in the liver. Liver sections were obtained from (**A**,**B**) noninfected mice or (**C**–**H**) mice infected with *M. avium* for 60 days. Sections were stained for (**A**–**D**,**G**,**H**) ferroportin, using immunohistochemistry (brown staining) or for (**E**,**F**) iron, using the Perls’ method (blue staining). (**E**–**H**) Mice were intraperitoneally injected with 10 mg of iron, as iron–dextran, 10 days before infection. Representative images of at least three animals per experimental condition. Representative granulomas are outlined. Scale bar: 200 µm.

In order to further understand how these alterations in gene expression translated into iron redistribution within the tissue, we performed a histological analysis of liver sections (Figure 5).

When noninfected liver sections were stained for ferroportin, we did not detect any differences in the protein distribution between *Fth1*^+/+^ and *Fth1*^−/−^ mice (Figure 5A,B). However, ferroportin distribution was markedly different between *Fth1*^+/+^ and *Fth1*^−/−^ mice during infection. While in *Fth1*^+/+^ mice, most ferroportin staining was found in hepatocytes (Figure 5C), in *Fth1*^−/−^ mice, most ferroportin was found inside granulomas (Figure 5D). Interestingly, this ferroportin distribution pattern nicely and inversely correlated with iron distribution, where iron was found predominantly within granulomas in infected *Fth1*^+/+^ mice (Figure 3I). However, evaluation of iron distribution by Perls’ staining is difficult in basal conditions, due to the low iron accumulation. To confirm the differences in iron and ferroportin distribution between *Fth1*^+/+^ and *Fth1*^−/−^ mice, we made a similar analysis in liver sections obtained from iron-overloaded mice. Again, in normal (*Fth1*^+/+^) mice, infection was accompanied by iron accumulation inside granulomas (Figure 5E), while most ferroportin staining was found in hepatocytes (Figure 5G). In contrast, in *Fth1*^−/−^ mice, the majority of iron was found in the liver parenchyma (Figure 5F) and most ferroportin staining was associated with granulomatous lesions (Figure 5H).

In summary, our data indicate that in the absence of FTH1 inside myeloid cells, iron does not accumulate inside this type of cells, but is actively exported by ferroportin and taken up by neighboring cells. In the context of the systemic infection with *M. avium*, the granulomatous lesions become devoid of iron, and this leads to limitation of bacterial growth, resulting in protection for the host and growth restriction for the bacteria.

## 3. Discussion

In this work, we show that FTH1 produced by myeloid cells plays a crucial role in iron redistribution during infection, being essential for iron deposition and storage inside macrophages. In the absence of myeloid-derived FTH1, a high ferroportin expression in macrophages leads to iron export by these cells and accumulation in the tissue parenchyma, namely hepatocytes. Upon infection by the intramacrophagic pathogen *M. avium*, the alteration in the iron distribution pattern associated with FTH1 deficiency in myeloid cells leads to increased host protection, due to decreased iron availability for bacterial proliferation. 

We and others previously demonstrated that iron availability is a crucial factor for the modulation of mycobacterial growth. The addition of iron chelators or the administration of a low-iron diet decrease the growth of mycobacteria, both in in vitro macrophage cultures and in vivo [22]. Conversely, increased *M. avium* growth is observed in mice with iron overload, resulting from experimental administration of iron or genetic abnormalities [23,24,25]. Deficiency in FTH1 leads to a decrease in intracellular iron in macrophages [3]. Thus, it is conceivable that in the current study, the slower growth of *M. avium* in *Fth1*^−/−^ mice was related to a decrease in iron availability inside the mycobacterial host cell, the macrophage. Reinforcing this idea, mice died with similar bacterial loads, indicating that the main difference between *Fth1*^−/−^ and *Fth1*^+/+^ mice was the rate at which bacteria were proliferating. Additionally, the lower bacterial loads in the liver and spleen of *Fth1*^−/−^ mice were reversed when the mice were iron-overloaded prior to infection. This interpretation may, however, be too simplistic, as the animal iron status has profound connections with other homeostasis circuits and, particularly, with the inflammatory response, as discussed below.

Our data are in clear contrast with Reddy et al., who showed that conditional deletion of *Fth1* in the myeloid lineage leads to increased mouse susceptibility to another mycobacterial species, *M. tuberculosis*. In that study, *Fth1* deletion in the myeloid lineage was associated, not only with higher bacterial loads, but also with an exacerbated inflammatory response, including larger lung lesions, splenomegaly, and loss of blood–brain barrier function [18]. Furthermore, myeloid *Fth1*^−/−^ mice infected with *M. tuberculosis* had increased levels of proinflammatory cytokines, including TNF-alpha and IFN-gamma, as compared to *Fth1*^+/+^ animals, which is in clear contrast with the decreased production of these cytokines that we observed in mice infected with *M. avium*. We could hypothesize that these marked differences between our study and that of Reddy et al. could be due to the different routes of infection. However, when we infected mice with *M. avium* by the aerosol route, *Fth1* deficiency was still associated with lower bacterial loads than in *Fth1*-sufficient mice. Furthermore, we performed an exploratory experiment with *M. tuberculosis*, in which we observed a tendency towards increased bacterial load in *Fth1*^−/−^ mice (unpublished). Overall, these observations indicate that the contrasting results obtained in infections by *M. avium* and *M. tuberculosis* are due to intrinsic characteristics of the two mycobacterial species, and not to any technical artifacts or confounding factors. In fact, differences between the two mycobacterial species have been observed in other animal models of infection, namely, mice deficient in inducible nitric oxide synthase, which are more susceptible to *M. tuberculosis* and more resistant to *M. avium* [26,27]. We can speculate that *M. avium* and *M. tuberculosis* also differ in the way they gain access to iron inside the tissues of the host.

*Fth1^−/−^* mice were also recently described to be more susceptible to infection by *Salmonella* [28]. Of note, in this work by Haschka et al., *Fth1^−/−^* mice were more susceptible to *Salmonella* only after iron overload. In this experimental condition, infection was associated with exacerbated inflammatory response, including increased circulating levels of TNFa, IL-6, and IL-1beta [28]. Apart from the important detail that differences in susceptibility between *Fth1*^+/+^ and *Fth1*^−/−^ mice were seen only after iron overload, these results are reminiscent of those obtained with *M. tuberculosis.* Both strongly suggest that in the absence of myeloid H-ferritin, increased iron-related oxidative stress leads to defects in the control of the inflammatory response with potential deleterious consequences for the host. This effect is, however, dependent on the pathogen involved. Our data are more in agreement with the study by Zarjou et al. in a model of sepsis induced by cecal ligation and puncture. Here, mice deficient in *Fth1* in the myeloid lineage had longer survival, lower organ damage, and lower levels of inflammatory cytokines compared to normal, *Fth1*-expressing animals, when sepsis was induced [19]. 

Interestingly, both in this sepsis study and in ours, no major alterations were observed in immune cell populations in lymphoid organs [19]. This is surprising, given the need for iron of proliferating lymphocytes and the suggestion that activated lymphocytes can bind H-ferritin [29]. It will be interesting to detail in future experiments the impact of iron redistribution caused by infection, in the presence or absence of myeloid H-ferritin, in the proliferation of different populations of lymphocytes. In the particular case of *M. avium* infection, the only significant difference we observed in spleen cell populations between *Fth1*^−/−^ and *Fth1*^+/+^ mice was the higher number of B cells in the former. We believe this difference is not relevant to explain the increased resistance to *M. avium* infection, as B cells were never shown to play a significant role in host protection in this model [30].

Zarjou et al. attributed the increased resistance of *Fth1*^−/−^ mice to sepsis to the compensatory increase in FTL expression, which could act as an iron scavenger and cell protection agent [19]. Consistent with this claim, a different study showed that mice deficient in *Fth1* in the myeloid lineage had a lower inflammatory response and better glucose tolerance and insulin sensitivity than *Fth1*^+/+^ mice after administration of a high fat diet [31]. In both these studies, as well as in the present work, the absence of FTH1 in myeloid cells and the consequent decrease of intracellular iron was associated with cell protection and increased survival. We cannot exclude that increased resistance to *M. avium* is also attributable to increased FTL levels present in the circulation and the tissues, but that was not directly addressed in our experiments. It is interesting to note that bone marrow-derived macrophages (BMM) derived in vitro from *Fth1*^−/−^ mice have lower intracellular iron levels and lower production of nitric oxide in response to LPS than *Fth1*^+/+^ controls, without a significant increase in the levels of FTL [3]. This suggests that the decreased intramacrophagic iron levels and decreased inflammatory response are independent of macrophage FTL expression. However, additional experiments with FTL supplementation in vivo would be necessary to clarify the contribution of this protein to the observed phenotype.

The lack of redundancy between FTH and FTL is well known, as well as the essentiality of FTH, linked to its catalytic activity [6,7]. However, several aspects of the differential functions of the two peptides are still unclear and are a fascinating field of research [10]. An increase in circulating ferritin is observed in several situations of infection and inflammation [14,15,32]. However, the cellular sources and export pathways of serum ferritin remain to be elucidated. Some authors defend that intracellular ferritin is passively released by cell death during inflammation [33,34], but abundant experimental evidence indicates that it can be actively released by different cell types, and several possible mechanisms were described [15,35,36,37,38]. The dogma in the field is that serum ferritin is composed exclusively of L chains [14,15]. However, in this work, we show that a significant proportion of circulating ferritin in *M. avium*-infected mice is composed of H chains. Furthermore, this high level of FTH1 in the serum was not observed in *Fth1*^−/−^ mice, indicating that it has a myeloid origin, in line with previous studies indicating that macrophages are the main source of circulating ferritin [35]. Interestingly, we and others previously showed that BMM increase the production of FTH1 when infected with *M. avium* or treated with TLR agonists in vitro, while the levels of FTL remain almost unaltered [16]. In fact, it is known that the expression of FTH is more sensitive to microbial or inflammatory stimuli than FTL (reviewed in [39]). Overall, it is thus plausible that the increased FTH1 production by macrophages during (mycobacterial) infection results in the release of H-chain-containing ferritin to the circulation. It would be interesting to investigate the physiological implications of this phenomenon.

FTH1 released from macrophages during infection may play an important role in systemic and local iron redistribution. It is generally accepted that during infections, iron accumulates inside macrophages. In the case of the mouse model of systemic infection by *M. avium*, we previously showed that iron tends to accumulate inside tissue granulomas [21,23]. This is true even in the case of the *Hfe*-knockout mouse, a model of hereditary hemochromatosis characterized by iron accumulation in parenchymal and not in myeloid cells [23]. This preferential accumulation of iron inside macrophages during mycobacterial infection was presumed to result from a downregulation of the expression of ferroportin in macrophages, either directly induced by microbial or immune mediators or through the activity of hepcidin [13,21,40]. Interestingly, in the present work, we show that iron accumulation inside granulomas depends on the expression of *Fth1* in myeloid cells. In the absence of this protein, there is an increase in the expression of ferroportin in granulomas and iron accumulates in the tissue parenchyma (Figure 5). We had previously shown that BMM obtained from *Fth1^−/−^* mice express higher levels of ferroportin in response to IFN-gamma and LPS or iron than *Fth1*^+/+^ BMM [3]. It is interesting to observe that this higher ferroportin expression prevails in vivo, during chronic infection by *M. avium*, surpassing possible downregulating signals. We cannot assume that the high ferroportin staining observed in granulomas is exclusively associated with macrophages. Other hematopoietic cells are present in these lesions and may contribute to the overall iron distribution in the tissue. However, macrophage–hepatocyte communication is particularly interesting for systemic iron homeostasis.

Macrophages play a central role in iron recycling, due to their erythrocyte removal function. Although in basal conditions, most erythrophagocytosis takes place in the spleen, several pathological conditions are associated with increased erythrophagocytosis in the liver [41]. We previously showed that during systemic infection of mice with *M. avium*, there is a marked increase of erythrocyte destruction in the liver, with a consequent increase in heme release and induction of heme-oxygenase-1 [20,42]. In fact, mice deficient in the *Hmox1* gene show a dramatic increase in susceptibility to *M. avium* and *M. tuberculosis* infections, related to increased oxidative stress and macrophage death [20]. The accumulation of iron inside granulomatous lesions, that we report here and in previous publications [21,23], is consistent with this scenario of increased iron turnover in the liver. However, what we found in this work is that iron accumulation inside granulomatous lesions depends on the availability of FTH1 in myeloid cells to provide iron storage conditions. In its absence, macrophages will readily export the iron resulting from erythrocyte destruction, which will remain in circulation at higher levels than under normal circumstances and will also be taken up by parenchymal cells. 

In this work, we present several new observations. We show that FTH1 in myeloid cells is required for iron retention in response to infection. In its absence, macrophages promptly release iron through increased expression of ferroportin, and iron accumulates predominantly inside parenchymal cells. We also show that in normal mice, a significant proportion of circulating ferritin during *M. avium* infection is composed of FTH1 chains derived from myeloid cells. Finally, we show that mice deficient in FTH1 in myeloid cells are more resistant to infection by *M. avium*, mainly due to a slower growth of the bacteria.

The mouse systemic *M. avium* infection offers a fascinating model to continue to explore the role of iron in the host–pathogen interaction.

## 4. Materials and Methods

### 4.1. Chemicals 

All chemicals were obtained from Sigma-Aldrich (St. Louis, MO, USA), unless otherwise specified.

### 4.2. Animals

Mice referred to as *Fth1^−/−^* mice are conditional *Fth1* deficient (*Fth1^Fl/^Fl*; *Lyz2^cre/+^*) mice, obtained by crossing *Fth1^Fl/Fl^* mice with *Lyz2^cre/+^* mice. An initial breeding pair was kindly provided by Prof. Lukas Kuhn (Swiss Institute for Experimental Cancer Research, Lausanne, Switzerland). In these mice, Cre recombinase deletes the *Fth1* gene in cells expressing *Lyz2* (cells of the myeloid lineage). *Fth1^Fl/Fl^; Lyz2^+/+^ Cre*- negative littermate mice were used as controls and are referred to as *Fth1^+/+^* mice. The mice were kept inside individually ventilated cages with HEPA filters and fed with sterilized food and water ad libitum. 

### 4.3. Bacteria 

*Mycobacterium avium* 25291 (obtained from the American Type Culture Collection, Manassas, VA, USA) and *Mycobacterium avium* 2447 SmT (*M. avium* strain 2447 smooth transparent variant (SmT), originally isolated by F. Portaels (Institute of Tropical Medicine, Antwerp, Belgium) from an AIDS patient, were grown at 37 °C in Middlebrook 7H9 broth (BD Difco, Franklin Lakes, NJ, USA), supplemented with 0.05% of Tween 80 and 10% albumin–dextrose–catalase. Mycobacteria were harvested during the exponential growth phase, centrifuged, washed twice with saline solution containing 0.04% Tween 80, resuspended in the same solution, and briefly sonicated at low power to disrupt bacteria clumps. Aliquots were prepared and stored at −80 °C until needed. Immediately before use, an aliquot was thawed and diluted to the appropriate concentration.

### 4.4. In Vivo Infection

Adult mice were infected with 1 million CFU of *M. avium* i.v. into one of the lateral tail veins. Control groups were injected with the same volume of saline solution by the same route. Sixty days after infection, mice were anesthetized in an isoflurane chamber. Blood was collected by retro-orbital puncture under anesthesia. Organs such as the liver and spleen were collected aseptically and weighted. Long bones were also collected. In iron overload experiments, 10 mg/animal of iron dextran was intraperitoneally administered 10 days before infection. Control animals were treated with the same volume of saline (numerous previous experiments carried out in our group indicated that there were no significant differences between mice injected with saline or injected with iron-free dextran in the amounts corresponding to this iron overload protocol [21,23,24]). For aerosol experiment, adult mice were infected with *M. avium* 25291 using an inhalation exposure system (Glas-Col, Terre Haute, IN, USA), to deliver a low infection dose (~100 CFU) to the lung of each mouse, as determined 3 days post-infection.

### 4.5. Quantification of Serum Cytokines

Cytokines’ quantification was performed using the BD cytometric bead array (CBA) Mouse Inflammation Kit (catalog no. 552364, BD Biosciences), which allows the simultaneous measurement of multiple cytokines. Serum samples were mixed with phycoerythrin detection reagent. The mixture was incubated for 2 h at RT in the dark. The samples were washed, the supernatant was discarded, and the beads were resuspended in washing buffer. Samples were then analyzed by using the BD FACSCanto II Bioanalyzer and FCAP Array software (BD Biosciences) according to the manufacturer’s instructions.

### 4.6. Serum Iron Parameters

Blood samples were collected by retro-orbital puncture under profound anesthesia and serum was obtained by high-speed centrifugation. Serum iron parameters were blindly determined in a certified laboratory (CoreLab, Centro Hospitalar do Porto, Porto, Portugal). The analysis was performed using a Sysmex XE-5000 hematology analyzer. 

### 4.7. ELISA

FTH1 and FTL were quantified by ELISA in the mice serum samples, using rabbit polyclonal anti-mouse FTH1 or FTL (DIBIT-IRCCS San Raffaele Scientific Institute, Milan, Italy) as primary antibodies, and biotinylated rabbit polyclonal anti-mouse FTH1 or FTL as the secondary antibodies. The specificity and absence of cross-reactivity of the antibodies have been previously described [19].

### 4.8. Iron Quantification

The determination of iron in tissues was performed by atomic absorption spectrometry. For this purpose, 100–180 mg of fresh tissue was dried at 65 °C to constant weight and then digested with 69% nitric acid. Measurements were performed on an AAnalyst 200 (PerkinElmer, Waltham, MA, USA) flame atomic absorption spectrometer, with an iron hollow cathode lamp (SCP Science, Quebec, Canada) as the radiation source. Absorbance was read at 248.33 nm in an air (10 L/min)–acetylene (2.5 L/min) flame. 

### 4.9. Analysis of Gene Expression

To extract RNA from liver samples, we used PureLink^®^ RNA Mini Kit, following the manufacturer’s instructions (Ambion^TM^, Invitrogen, Carlsbad, CA, USA). Briefly, lysis buffer, supplemented with 1% β-mercaptoethanol, was added to a liver piece and homogenized. The centrifuged supernatant was transferred to a new tube and the same volume of ethanol 70% was added. After mixing, the whole volume was transferred to the mini-columns. The extracted RNA was eluted with RNAse-free water from the column to a proper tube. The samples were stored at −80 °C, prior to conversion to cDNA and qPCR analysis. Total RNA was transcribed into cDNA by using an NZY First-Strand cDNA Synthesis Kit (NZYTech, Lisbon, Portugal). For the amplification of each gene of interest, a corresponding specific pair of primers (STAB Vida, Lisbon, Portugal) was used (Table 2). All reactions were performed in a 20 μL total volume with iTaq SYBR green PCR master mix (Bio-Rad, Hercules, CA, USA). Baseline thresholds were obtained by using the Bio-Rad iQ5 program, and the threshold cycles (CTs) were calculated by the 2CT method, where CT values for the genes of interest were normalized to the level of the hypoxanthine-guanine phosphoribosyl transferase housekeeping gene (*Hprt1*). Data are shown as n-fold differences relative to values for noninfected samples of *Fth1*^+/+^ genotype, calculated with the 2^−ΔΔCT^ method. 

### 4.10. Histological Analysis

Liver samples were fixed in a 10% neutral buffered formalin solution and included in paraffin blocks prior to hematoxylin and eosin (H&E) and Perls’ blue staining. Three-micrometer-thick paraffin sections were deparaffinized and processed through downgraded alcohols. For H&E staining, sections were rehydrated and stained with hematoxylin solution modified according to Gill III (Merck Millipore, Darmstadt, Germany) for nuclear staining and rinsed in 0.1% HCl solution: 37% HCl and distilled water. Sections were differentiated under running water. Counterstaining of proteins, collagen, keratin, or connective tissue was performed by staining of tissue sections with aqueous eosin Y solution 0.5%. For the detection of ferric iron, sections were stained by the Perls’ method. Briefly, sections were rehydrated, and incubated in a 2% potassium ferrocyanide trihydrate–2% HCl solution for 30 min. Sections were rinsed three times with water and counterstained with filtered neutral red for 10 min. Then, sections were rinsed and rapidly dehydrated (reverse of downgraded alcohols and xylene), and coverslipped in Entellan^TM^. Sections were imaged in NanoZoomer 2.0 HT (Hamamatsu Photonics, Hamamatsu City, Japan) with a magnification of 40× and a resolution of 0.226 µm. The slides were imported to QuPath (version 0.3) and, following image annotation, a random forest pixel classifier was trained to segment the tissue and granulomas. The percentage of granuloma area was calculated as Granuloma % = Granuloma/(Granuloma + Tissue) × 100%, where Granuloma represents the total area classified as granuloma and tissue represents the total area classified as tissue parenchyma. Representative images were obtained with Olympus Cx31 microscope (Olympus Lifesciences, Tokyo, Japan (objective 10×), Michrome 5 Pro camera (Tucsen Photonics, Fuzhou, Fujian, PRC), and Mosaic 2.3 software.

### 4.11. Immunohistochemistry for Ferroportin

Sections were deparaffinized in two changes of xylene and hydrated using decreasing percentage of alcohol chambers before being rinsed in water. To unmask the antigenic epitope, sections were incubated in citrate buffer (pH 6) (Epredia™Lab Vision™ HIER Buffer L, Kalamazoo, MI, USA) with 0.05% Tween 20 in a steamer for 40 min and allowed to cool for 20 min at RT and washed twice with TBS-T. In a humidified chamber, endogenous peroxidase activity was blocked using Peroxidase (Epredia^TM^ UV hydrogen peroxide block) solution for 10 min. Nonspecific binding of secondary antibodies/reagents was prevented using antibody blocker (Enzo Life Sciences, Farmingdale, NY, USA) for 10 min. After washing, sections were incubated with anti-ferroportin antibody (SLC40A1 from Novus Biologicals, Bio-Techne Ltd., Abingdon, UK) antibody at 1:1000 overnight, at 4 °C. Then, after washing again with TBS-T, the secondary antibody Poliview^®^ Plus HRP anti-rabbit (Enzo Life Sciences) was added for 45 min. Finally, DAB solution was applied to allow color development. Slides were counterstained with Hematoxylin QS (Vector Laboratories, Burlingame, CA, USA) for 20 s and differentiated in tap water for 5–10 min. Slides were dehydrated through four changes of alcohol (95%, 95%, 100%, and 100%), cleared, and coverslipped using Entellan^TM^. The sections were imaged in D-Sight Plus f2.0 and analyzed with D-Sight viewer (both from A. Menarini Diagnostics).

### 4.12. Cell Population Characterization

Spleens were dissociated to a single-cell suspension and then filtered using a 70 µm nylon mesh. Cells were counted using trypan blue exclusion assay. A number of 1 × 10^6^ cells were plated and stained with anti-F4/80 (1:100, cloneBM8; BioLegend, San Diego, CA, USA), CD169 (1:100, clone 3D6.112; BioLegend), CD115 (1:100, clone 3D6.112; BioLegend), Gr1 (1:500, clone RB6-8C5; BioLegend), CD19 (1:100, clone CD5, BioLegend), CD4 (1:100, BM4-5, BD Horizon, BD Biosciences, San Jose, CA, USA), CD8 (1:200, clone 53-6.7, BioLegend), and NK1.1 (1:100, clone Pk136 BioLegend) for 30 min, at 4 °C. Then, cells were washed and fixed with 2% PFA for 10 min at room temperature (RT). Cells were washed and analyzed using a BD FACS Canto II flow cytometer and FlowJo (BD Biosciences). 

### 4.13. Western Blot

Equivalent amounts of protein prepared in Laemmli buffer were separated by electrophoresis as previously described [3]. PVDF membranes were blocked, followed by overnight incubation with the primary polyclonal antibodies H- and L-ferritin subunits [43] (FTH1, 1:1000 from Cell Signalling; FTL, 1:1000 from Abcam) prepared in 1% bovine serum albumin (BSA)/TBS-T. The membranes were washed with TBS-T, and incubated with the secondary antibody (anti-rabbit, 1:10,000, The Binding Site, Birmingham, UK) in 1% BSA/TBS-T for 1 h at RT. Loading control was performed with Ponceau staining. The imaging of the membranes was made using ChemiDoc (Bio-Rad, Hercules, CA, USA), with the help of horseradish peroxidase (HRP) (Luminata^TM^ Milipore, Billerica, MA, USA) substrate. 

### 4.14. Statistical Analysis 

Data analysis was performed using GraphPad Prism 8.0 program (GraphPad Software, Inc., La Jolla, CA, USA), and data were expressed as means ± standard deviation or by bars (mean) together with circles representing each individual value. The number of samples is indicated in the legend of each figure. Multiple comparisons were performed by using two-way-ANOVA followed by a post hoc Sidak test. Comparisons between two groups were performed by using the unpaired Student’s *t* test. In both cases, differences were considered significant when the *p* value was < 0.05.

## Figures and Tables

**Table 1:** Iron-related gene expression induced by mycobacterial infection.

	Noninfected		Infected	
Gene	*Fth1* ^+/+^	*Fth1* ^−/−^	*Fth1* ^+/+^	*Fth1* ^−/−^
*Fth1*	1.01 ± 0.09	1.01 ± 0.42	6.73 ± 1.64	0.54 ± 0.02
*Ftl*	1.00 ± 0.05	1.01 ± 0.53	1.70 ± 0.35	0.76 ± 0.09
*Hmox*	1.15 ± 0.33	0.88 ± 0.21	124.1 ± 51.51 ^#^	46.27 ± 20.28 ^#^
*Hamp1*	1.03 ± 0.11	1.43 ± 0.24	191.70 ± 41.45 ^###^	88.70 ± 6.28 ^#^
*Slc40a1*	1.04 ± 0.07	0.97 ± 0.47	3.55 ± 2.20	2.09 ± 0.45

Data represent average ± SD of fold change relative to noninfected *Fth1*^+/+^ mice, from 3–6 animals. ^###^ *p* < 0.001, ^#^ *p* < 0.05 when comparing infected with noninfected mice within the same genotype.

**Table 2:** Primers sequences.

Gene and Nomenclature	Primer	Sequence
*Hypoxanthine Phosphoribosyltransferase (Hprt)*	*Hprt forward*	5′-GGTGGAGATGATCTCTCAAC-3′
	*Hprt reverse*	5′-TCATTATAGTCAAGGGCATATCC-3′
*H-ferritin (Fth1)*	*Fth1 forward*	5′-GCTGAATGCAATGGAGTGTGCA-3′
	*Fth1 reverse*	5′-GGCACCCATCTTGCGTAAGTTG-3′
*L-ferritin (Ftl)*	*Ftl forward*	5′-ACCTACCTCTCTCTGGGCTT-3′
	*Ftl reverse*	5′-TGGCTTCTGCACATCCTGGA-3′
*Ferroportin (Slc40a1)*	*Slc40a1 forward*	5′-TTGGTGACTGGGTGGATAAGAATGC-3′
	*Slc40a1 reverse*	5′-CGCAGAGGATGACGGACACATTC-3′
*Heme oxygenase 1 (Hmox1)*	*Hmox1 forward*	5′-GCCACCAAGGAGGTACACAT-3′
	*Hmox1 reverse*	5′-GCTTGTTGCCCTCTATCTCC-3′
*Hepcidin (Hamp1)*	*Hamp1 forward*	5′-CCTATCTCCATCAACAGATG-3′
	*Hamp1 reverse*	5′-AACAGATACCACACTGGGAA-3′

## Data Availability

All data generated in this study is included in the manuscript.

## Supplementary Materials

The following are available online at https://www.mdpi.com/article/10.3390/ijms23010269/s1.
