# Supplementary material for: H-Ferritin Produced by Myeloid Cells Is Released to the Circulation and Plays a Major Role in Liver Iron Distribution during Infection
Source: Int J Mol Sci. 2021 Dec 27;23(1):269. doi: 10.3390/ijms23010269 (PMC8745395; doi:10.3390/ijms23010269)
Supplement: Supplementary file 1 [file ijms-23-00269-s001.zip › ijms-1500492-supplementary2.0.pdf]

SUPPLEMENTARY INFORMATION

H-ferritin produced by myeloid cells is released to the circulation and plays a major role in liver iron distribution during infection

Ana C. Moreira [1,2,3,†], Tânia Silva [1,2,3,†], Gonçalo Mesquita [2,‡], Ana Cordeiro Gomes [1,2], Clara M. Bento [1,2,4], João V. Neves [1,2,3], Daniela F. Rodrigues [1,2], Pedro N. Rodrigues [1,2,3], Agostinho A. Almeida [5], Paolo Santambrogio [6] and Maria Salomé Gomes [1,2,3,*]

[1] i3S—Instituto de Investigação e Inovação em Saúde, Universidade do Porto, 4200-135 Porto, Portugal; Ana.S.Moreira@ibmc.up.pt (A.C.M.); tania.silva@ibmc.up.pt (T.S.); ana.c.gomes@i3s.up.pt (A.C.G.); clara.bento@i3s.up.pt (C.M.B.); jneves@ibmc.up.pt (J.V.N.); drodrigues@ibmc.up.pt (D.F.R.); prodrigu@ibmc.up.pt (P.N.R.)

[2] IBMC—Instituto de Biologia Molecular e Celular, Universidade do Porto, 4200-135 Porto, Portugal; goncalo.mesquita@univ-lille.fr

[3] ICBAS—Instituto de Ciências Biomédicas Abel Salazar, Universidade do Porto, 4050-313 Porto, Portugal

[4] Programa Doutoral em Biologia Molecular e Celular (MCbiology), Instituto de Ciências Biomédicas Abel Salazar da Universidade do Porto, 4200-135 Porto, Portugal

[5] LAQV/REQUIMTE, Departamento de Ciências Químicas, Faculdade de Farmácia, Universidade do Porto, 4050-313 Porto, Portugal; aalmeida@ff.up.pt

[6] Division of Neuroscience, San Raffaele Scientific Institute, 20132 Milan, Italy; santambrogio.paolo@hsr.it

* Correspondence: sgomes@ibmc.up.pt

† These authors contributed equally to this work.

‡ Current address: Laboratory of Cell Physiology, INSERM U1003, Laboratory of Excellence Ion Channels Science and Therapeutics, Department of Biology, Faculty of Science and Technologies, University of Lille, 59650 Villeneuve d'Ascq, France; PHYCELL-Laboratoire de Physiologie Cellulaire, INSERM U1003,
University of Lille, 59655 Villeneuve d'Ascq, France.

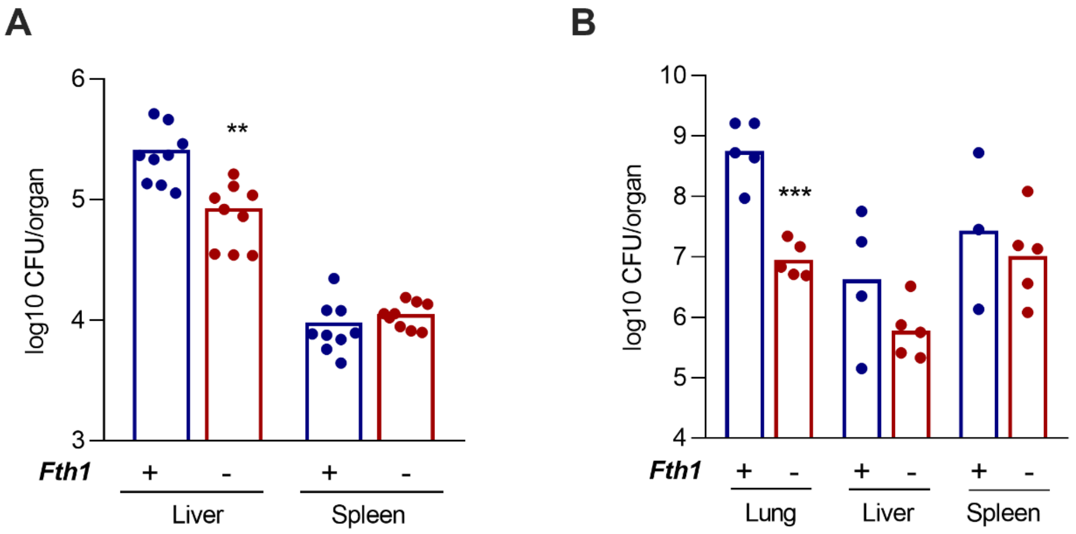

Figure S1. Mice deficient in H-ferritin in the myeloid cells are more resistant in different models of *M. avium* infection. (**a**) *Fth1*$^{+/+}$ (blue) and *Fth1*$^{-/-}$ (red) mice were intravenously infected with 10^6 CFU of *M. avium* 2447 SmT. Bacterial burden in the liver and spleen, 60 days post infection (N=9). (**b**) *Fth1*$^{+/+}$ (blue) and *Fth1*$^{-/-}$ (red) mice were infected via aerosol with a low dose (~100 CFU/lung) of *M. avium* 25291. Bacterial burden in the lung, liver and spleen, 125 days post infection. (N=4 to 5). Bars represent the mean and the circles represent each animal. Statistics: multiple t-test. **P<0.01, ***P<0.001 when comparing between genotypes.

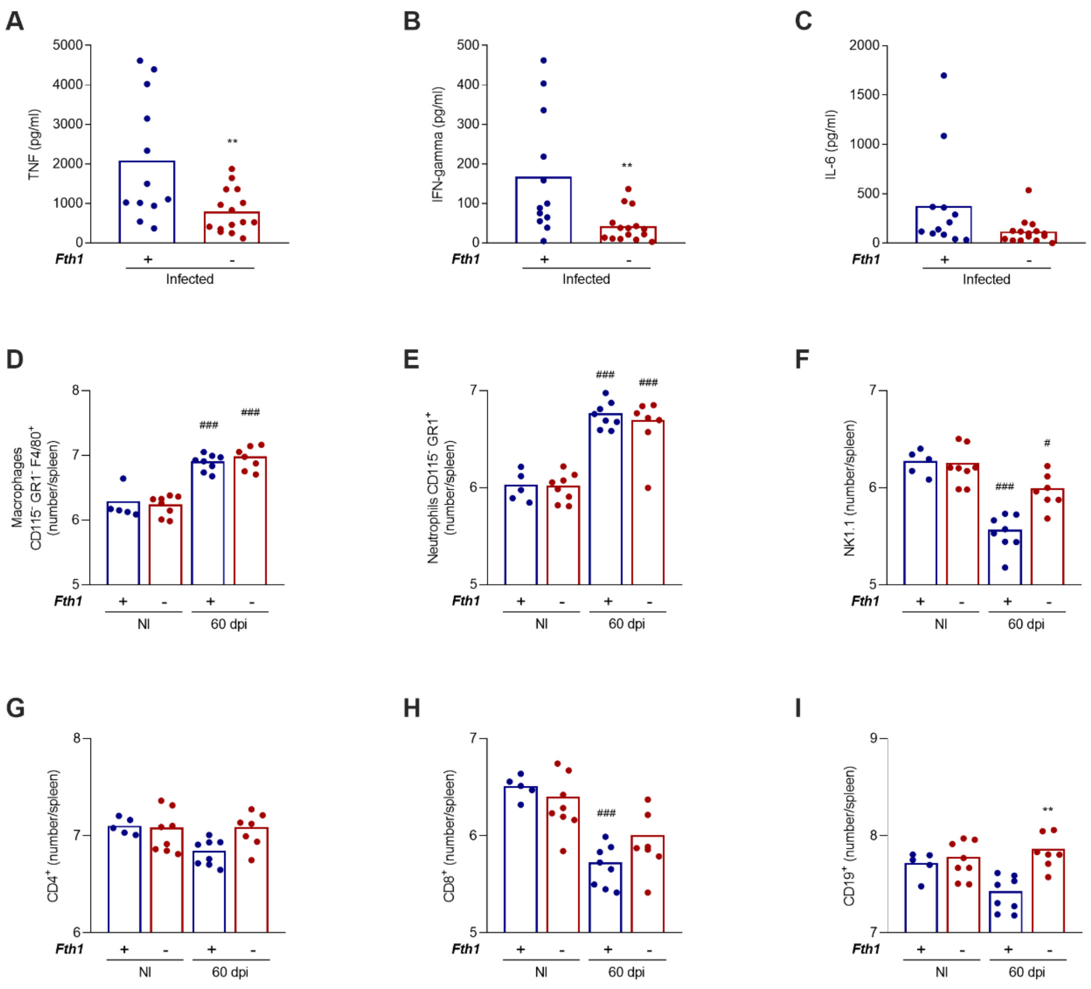

Figure S2. *Fth1*$^{-/-}$ mice have lower levels of inflammatory cytokines in circulation and spleen cell populations are not dependent of FTH1 expression. (**a**) TNF-alpha, (**b**) IFN-gamma, (**c**) IL-6 were measured in the serum of the mice from the survival curve, using a cytometric bead array mouse inflammation kit (N = 12 (*Fth1*$^{+/+}$) or 16 (*Fth1*$^{-/-}$)). (**d-i**) Number of macrophages (GR1-F4/80+), neutrophils (CD115-GR1+), NK1.1, CD4+, CD8+, CD19+ CD19+, NK1.1, CF4+, CD8+ cells in the spleen 60 days post infection (N=5 to 8). Bars represent the mean and the circles represent each animal. Statistical analysis was performed by two-way ANOVA with Sidak multiple comparison post hoc test. **P<0.01 when comparing between genotypes, ###P<0.001 when assessing the effect of infection within the same genotype.

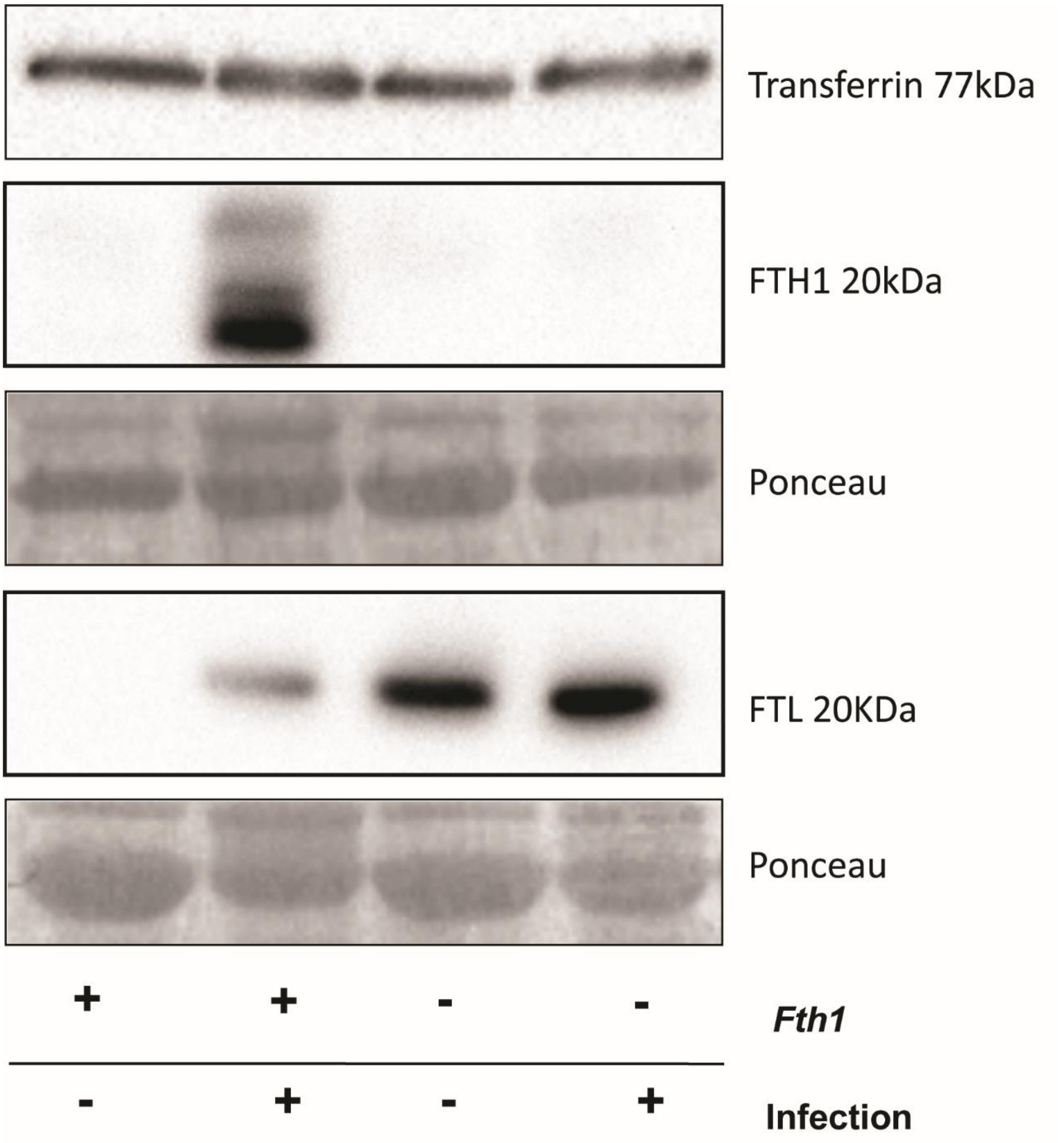

Figure S3. During *M. avium* infection, *Fth1*$^{+/+}$ mice have high amount of FTH1 in the serum. Western Blot against transferrin, FTH1 and FTL subunits. Ponceau staining was used to stain all proteins in the gel, as a confirmation that all lanes were loaded with equivalent amounts of protein.
